# Application of Bayesian regression with singular value decomposition method in association studies for sequence data

**DOI:** 10.1186/1753-6561-5-S9-S57

**Published:** 2011-11-29

**Authors:** Soonil Kwon, Xiaofei Yan, Jinrui Cui, Jie Yao, Kai Yang, Donald Tsiang, Xiaohui Li, Jerome I Rotter, Xiuqing Guo

**Affiliations:** 1 Medical Genetics Institute, Cedars-Sinai Medical Center, Los Angeles, CA 90048, USA; 2 Center for Biostatistics and Bioinformatics, Cedars-Sinai Medical Center, Los Angeles, CA 90048, USA; 3 Department of Biomedical Sciences, Cedars-Sinai Medical Center, Los Angeles, CA 90048, USA

## Abstract

Genetic association studies usually involve a large number of single-nucleotide polymorphisms (SNPs) (*k*) and a relative small sample size (*n*), which produces the situation that *k* is much greater than *n*. Because conventional statistical approaches are unable to deal with multiple SNPs simultaneously when *k* is much greater than *n*, single-SNP association studies have been used to identify genes involved in a disease’s pathophysiology, which causes a multiple testing problem. To evaluate the contribution of multiple SNPs simultaneously to disease traits when *k* is much greater than *n*, we developed the Bayesian regression with singular value decomposition (BRSVD) method. The method reduces the dimension of the design matrix from *k* to *n* by applying singular value decomposition to the design matrix. We evaluated the model using a Markov chain Monte Carlo simulation with Gibbs sampler constructed from the posterior densities driven by conjugate prior densities. Permutation was incorporated to generate empirical *p*-values. We applied the BRSVD method to the sequence data provided by Genetic Analysis Workshop 17 and found that the BRSVD method is a practical method that can be used to analyze sequence data in comparison to the single-SNP association test and the penalized regression method.

## Background

Association studies usually involve a large number of single-nucleotide polymorphisms (SNPs) (*k*) and a relatively small number of samples (*n*). To avoid multiple testing problems and to consider the effect of multiple SNPs simultaneously, investigators need statistical models that will test multiple SNPs simultaneously. Because standard statistical methods are unable to analyze multiple SNPs simultaneously when *k* is much greater than *n*, Tibshirani [1] introduced the penalized regression (PR) method as an alternative. The method reduces the size of SNP coefficients by treating the coefficients with little effect as zero. In other words, only those SNPs that significantly improve prediction are kept in the model. A potential drawback of this method is that a SNP with a strong marginal effect might be removed from the model if some other SNPs can explain the effect. A second drawback is that the number of SNPs evaluated in the model is controlled by the chosen penalization parameter. Even though the PR method does evaluate multiple SNPs simultaneously when *k* is much greater than *n*, the maximum number of SNPs that can be evaluated in the model is limited by sample size; that is, the method usually cannot test all SNPs simultaneously in large-scale genetic association studies, such as genome-wide association studies.

To evaluate all SNPs simultaneously in one statistical model, we introduced the Bayesian classification with singular value decomposition (BCSVD) method [2]. The BCSVD method can be applied to a dichotomous response variable when *k* is much greater than *n*. The method achieves a massive dimension reduction by applying singular value decomposition to the design matrix in a binary probit model; it estimates the effect of SNPs through the reduced model. Selection of significant SNPs can be achieved by using the empirical *p*-values obtained from permutation. The BCSVD method handles small sample sizes quite well.

To analyze quantitative traits when k is much greater than n, we further developed the Bayesian regression with singular value decomposition (BRSVD) method. We applied the BRSVD method to the sequence data provided by Genetic Analysis Workshop 17 (GAW17). We show that the BRSVD method is a practical method that can be used to analyze sequence data by comparison to the single-SNP association test and PR methods.

## Methods

### BRSVD method

Let us consider the standard regression model in the matrix form:(1)

where *y_n_*_×1_ is a vector of quantitative dependent variables, *X_n_*_×_*_k_* is the design matrix, *β_k_*_×1_ is a vector of parameters to be estimated, *I_n_* is an *n* × *n* identity matrix, and *σ*^2^ is an unknown variance; as before, *k* and *n* are the number of SNPs and the number of samples, respectively. By applying singular value decomposition (SVD) to the design matrix *X*′ = *ADF*′, the model in Eq. (1) with the SVD of *X* can be written:(2)

where *L* = *FD* and:(3)

As in Kwon et al. [2], we call *γ* a superfactor vector because it is expressed as a linear combination of the original parameters *β*. The statistical inference will be held on the superfactor vector instead of on *β.* From Eq. (2), the likelihood function of *y* given (*γ*, *σ*^2^) can be obtained as:(4)

where:(5)

and  is the maximum-likelihood (or least-squares) estimator of *γ.* Let us choose prior densities for (*β*|*σ*^2^) and *σ*^2^ as:(6)

and(7)

where *IG* is the inverted gamma distribution and (β*, *m*, *a*, *b*) are known hyperparameters. Because *γ* = *A*′*β*, the conjugate prior density on *β* implies the conjugate prior density on *γ* so that:(8)

Thus the prior density on (*γ*, *σ*^2^) can be expressed as:(9)

The joint posterior distribution for (*γ*, *σ*^2^) can be obtained by multiplying the likelihood function in Eq. (4) to the prior density in Eq. (9):(10)

where(11)(12)(13)(14)(15)

The marginal densities for *γ* and *σ*^2^ can be obtained by integrating Eq. (10) with respect to *σ*^2^ and *γ*, respectively. Given the observed data, the marginal posterior density for *γ* is a multivariate Student’s *t* distribution in which each element is a Student’s *t* distribution with (*n* + *a*) degrees of freedom and the marginal density for *σ*^2^ is:(16)

With these posterior distributions, the *γ* can be estimated through a Markov chain Monte Carlo simulation with Gibbs sampler, which starts with the maximum-likelihood estimate. To transform the superfactor vector (*γ*) in Eq. (2) back to *β*, which is our original parameter of interest vector, we use the most general solution form for the linear equation (*γ* = *A*′*β*) and achieve the unique solution for *β* by choosing the generalized inverse of *A*′ as *A *[3]. We use a permutation test to estimate the significance of the SNP effects on the phenotype. Let  be the estimate of the *i*th SNP effect from the raw data, and let  be the estimate of the *i*th SNP effect from the jth shuffled data that were obtained by permuting the quantitative trait (y). Define  as the difference between  and . Then the test statistic can be defined as:(17)

where  is the sample mean of  and  is the standard error of . Under the null hypothesis (*H*_0_: *β_i_* = 0), the statistic Λ*_i_* follows the standard normal distribution when *J* is large:(18)

### Study sample and association analysis

We used the unrelated individuals data distributed by GAW17, which includes 697 individuals, 24,487 SNPs, and 3 covariates (sex, age, and smoking status). We analyzed the first 10 replicates of phenotypes for quantitative risk factor Q1. We first performed the single-SNP association test using the simple linear regression model option in PLINK [4]. Second, we applied the PR method with L1 penalty introduced by Tibshirani [1] using the R package monomvn [5]. We evaluated SNP association with Q1 within the maximum number of SNPs allowed by the package in each step, which is min(*k*, *n* − intercept). Because the package does not provide *p*-values, we used the same permutation technique as in the BRSVD method to obtain empirical *p*-values. Third, we implemented the BRSVD method. To define significant SNPs for each method, we considered the following statistical models: quantitative risk factor Q1 versus the single SNP and the three covariates for the single-SNP association test; quantitative risk factor Q1 versus the maximum number of SNPs allowed by the package plus the three covariates for the PR method; and quantitative risk factor Q1 versus all SNPs (24,487) and the three covariates for the BRSVD method. All SNPs identified as significant for each model were compared to the 39 SNPs listed in the answer sheet distributed by GAW17. The analyses were run for each of the first 10 replicates, and the average of the 10 replicates was summarized (see Results section).

## Results and discussion

### Single-SNP association

**Figure 1:**
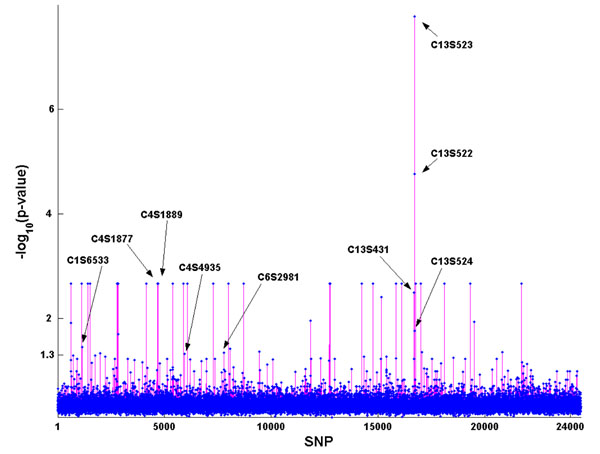
**Single-SNP association analysis from PLINK***. x*-axis: All SNPs on chromosomes 1–22 are numbered from 1 to 24,487. *y*-axis: −log_10_(*p*-value). The names for the two SNPs that were correctly identified are given.

Using a *p*-value less than 10^−5^, which is an approximate value of 0.05 genome-wide level using Bonferroni correction, as the cut point, the single-SNP test identified age and 50 SNPs as the risk factors for Q1 (Figure 1). By comparison with the answer sheet distributed by GAW17, which listed 39 SNPs that were associated with Q1, only 2 SNPs (C13S522 and C13S523) out of the 50 were correctly identified.

### PR method

**Figure 2:**
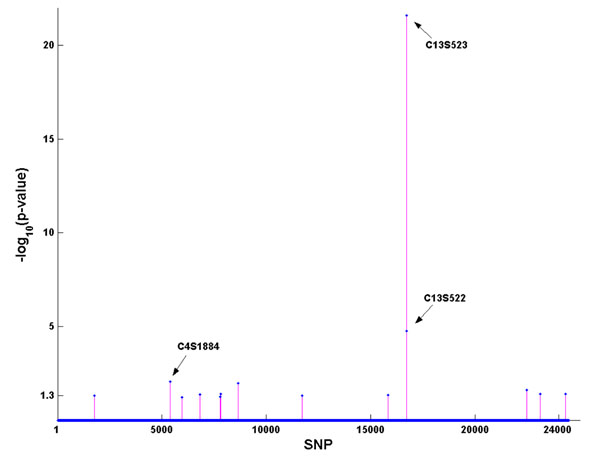
**Association results from the penalized regression method***. x*-axis: All SNPs on chromosomes 1–22 are numbered from 1 to 24,487. *y*-axis: −log_10_(*p*-value). The three correctly identified SNPs are given.

The results from the PR method are shown in Figure 2. With the cut point of *p* = 0.05 (i.e., −log_10_(*p*) = 1.3), age was again identified as a risk factor for Q1. In addition, 15 SNPs were also found to be significant. However, only 3 SNPs (C13S523, C13S522, and C4S1884) out of the 15 were on the 39 risk SNPs list.

### BRSVD method

**Figure 3:**
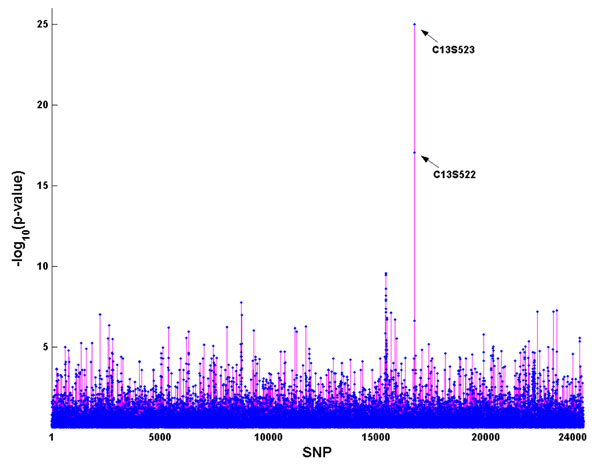
**Association results from the BRSVD method. ***x*-axis: All SNPs on chromosomes 1–22 are numbered from 1 to 24,487. *y*-axis: −log_10_(*p*-value). The nine correctly identified SNPs are given.

Figure 3 summarizes the association analysis results using the BRSVD method. We applied the cut point of *p* = 0.05 (i.e., −log_10_(*p*) = 1.3) because all SNPs were evaluated in one test. Age and 45 SNPs were found to be significant. Comparing to the 39 SNPs listed in the answer sheet, 9 SNPs (C1S6533, C4S1877, C4S1889, C4S4935, C6S2981, C13S431, C13S522, C13S523, and C13S524) out of the 45 SNPs were correctly identified.

Table 1 summarizes the validity of the three methods. The single-SNP association analysis method had a positive predictive value (PPV) of 4% compared to 18.7% for the PR method and 20% for the BRSVD method. We therefore concluded that the single-SNP association analysis method is less efficient than the other two methods for identifying the associated SNPs for the quantitative risk factor Q1. The BRSVD method is slightly better than the PR method, even though they performed almost at the same efficiency level of about 20%. Negative predictive values (NPVs) were greater than 99% for all three methods. This could be explained by the combination of (1) having a large number of SNPs, most of which are not associated with the disease, and (2) choosing a stringent selection criterion so that only a few SNPs are selected. The specificity showed that all three methods correctly classified more than 99% of the SNPs that are not significant. The false-positive rate (FPR = 1 − specificity) can therefore be calculated as 0.002 for single-SNP association, 0.0005 for the PR method, and 0.0015 for the BRSVD method. However, the sensitivity, which shows the power of analysis, demonstrates that the BRSVD method (23.1%) is considerably more powerful than the other two methods (5.1% for the single-SNP association test and 7.7% for the PR method).

**Table 1:** Summary of validation of the three methods

	Empirical outcome								
	
	Single-SNP association			PR method			BRSVD method		
	
Actual outcome	E′ (= 50)	IE′ (= 24,437)		E′ (= 16)	IE′ (= 24,471)		E′ (= 45)	IE′ (= 24,442)	
*E* (= 39)	TP = 2	FN = 37	Sen = 0.051	TP = 3	FN = 36	Sen = 0.077	TP = 9	FN = 30	Sen = 0.231
IE (= 24,448)	FP = 48	TN = 24,400	Spe = 0.998	FP = 13	TN = 24,435	Spe = 0.9995	FP = 36	TN = 24,412	Spe = 0.9985

	PPV = 0.04	NPV = 0.9984		PPV = 0.187	NPV = 0.9985		PPV = 0.2	NPV = 0.9988	

Empirical analysis results for the three methods. E is the number of SNPs that are truly effective, IE is the number of SNPs that are ineffective). E` is the number of SNPs that are empirically effective, IE is the number of SNPs that are ineffective); TP, true positive; FP, false positive; FN, false negative; TN, true negative; PPV, positive predictive value; NPV, negative predictive value; Sen, sensitivity; Spe, specificity.

## Conclusions

We used three different analysis methods (the single-SNP association analysis method implemented in PLINK, as widely used in genome-wide association studies; the PR method; and the BRSVD method) to identify SNPs that significantly influence the quantitative trait Q1 using the unrelated-individuals sample provided by GAW17. Both the PR and BRSVD methods out-performed the single-SNP association analysis method, suggesting that evaluating multiple SNPs simultaneously not only reduced the problems of multiple testing but also provided more power than single-SNP association in genetic association studies. The BRSVD method had a sensitivity almost three times as high as that of the PR method, suggesting that the BRSVD method is more optimal than the PR method. Another advantage of the BRSVD method is that it requires no specification of parameters compared to the PR method, which requires specification of the penalization parameter that controls the number of variables selected. Moreover, the BRSVD method takes much less computing time than the PR method does. For the association analysis of Q1 in the GAW17 unrelated individuals data, the PR methods used about 1.5 times as much run-time as the BRSVD method. With all factors considered, we believe that the BRSVD method is a good choice for large-scale genetic association study for quantitative traits.

## Competing interests

The authors declare that there are no competing interests.

## Authors’ contributions

SK participated in the design, analysis and drafted the manuscript. XY participated in data cleaning and initial data analysis. JC participated in GWAS analysis. JY helped with data analysis. DT helped to manage the data and analysis. XL helped with data analysis. JIR helped to draft the manuscript. XG participated in its design and coordination and helped to draft the manuscript. All authors read and approved the final manuscript.
